# Validating Community-Led Forest Biomass Assessments

**DOI:** 10.1371/journal.pone.0130529

**Published:** 2015-06-30

**Authors:** Michelle Venter, Oscar Venter, Will Edwards, Michael I. Bird

**Affiliations:** 1 School of Earth and Environmental Science, James Cook University, Cairns, Queensland, Australia; 2 Centre for Tropical Environmental and Sustainability Science, James Cook University, Cairns, Queensland, Australia; 3 School of Marine and Tropical Biology, James Cook University, Cairns, Queensland, Australia; 4 Center of Excellence for Environmental Decisions, University of Queensland, Brisbane, Australia; Chinese Academy of Forestry, CHINA

## Abstract

The lack of capacity to monitor forest carbon stocks in developing countries is undermining global efforts to reduce carbon emissions. Involving local people in monitoring forest carbon stocks could potentially address this capacity gap. This study conducts a complete expert remeasurement of community-led biomass inventories in remote tropical forests of Papua New Guinea. By fully remeasuring and isolating the effects of 4,481 field measurements, we demonstrate that programmes employing local people (non-experts) can produce forest monitoring data as reliable as those produced by scientists (experts). Overall, non-experts reported lower biomass estimates by an average of 9.1%, equivalent to 55.2 fewer tonnes of biomass ha^-1^, which could have important financial implications for communities. However, there were no significant differences between forest biomass estimates of expert and non-expert, nor were there significant differences in some of the components used to calculate these estimates, such as tree diameter at breast height (DBH), tree counts and plot surface area, but were significant differences between tree heights. At the landscape level, the greatest biomass discrepancies resulted from height measurements (41%) and, unexpectedly, a few large missing trees contributing to a third of the overall discrepancies. We show that 85% of the biomass discrepancies at the tree level were caused by measurement taken on large trees (DBH ≥50cm), even though they consisted of only 14% of the stems. We demonstrate that programmes that engage local people can provide high-quality forest carbon data that could help overcome barriers to reducing forest carbon emissions in developing countries. Nonetheless, community-based monitoring programmes should prioritise reducing errors in the field that lead to the most important discrepancies, notably; overcoming challenges to accurately measure large trees.

## Introduction

Potential exists to mitigate anthropogenic climate change by reducing the rates of forest carbon loss and increasing forest carbon sequestration [1]. Acknowledging this potential, the international community has developed mechanisms to reward efforts that halt and ultimately reverse forest carbon loss in developing countries [2]. To take part in these efforts, developing countries must first establish their own system for monitoring carbon stocks and fluxes from their forests [3].

For important carbon pools, such as those of tropical forests, the Intergovernmental Panel on Climate Change (IPCC) recommends a high level of accuracy in monitoring (Tier 3), attained through a combination of field and remote-sensing inventories [4]. However, field inventories are resource intensive because they require teams of experts to work, often in remote locations, for extended periods. For this reason, the capacity of most developing countries falls short of the requirements needed to monitor forest carbon stocks. Only 7 of the 99 non-Annex I countries in tropical regions can perform field-based forest biomass-carbon inventories [5]. Clearly, additional resources and more effective methods are needed.

One option for increasing the capacity of developing countries is to engage local people in monitoring forest carbon stocks [6]. Aside from contributing directly to the monitoring process, local participation can help improve natural resource management [7], the likelihood of permanence in emissions reductions and provide alternative livelihoods for those who forego destructive forest exploitation [8]. Additionally, these types of programmes could efficiently channel REDD+ (Reducing Emissions from Deforestation and Forest Degradation) incentives and rewards toward local communities [8]. For this reason, the potential for local people to contribute to the monitoring process has not gone unnoticed. Community forest monitoring was identified as a central component of national REDD+ readiness plans for multiple countries [9]. Fledgling programmes to harness this capacity are already in place in Tanzania, Nepal and Papua New Guinea [9]. Furthermore, the Subsidiary Body for Scientific and Technological Advice (SBSTA) recognises and promotes the engagement of Indigenous Peoples and local communities in monitoring and reporting activities [10, 11].

Given that community monitoring programmes are emerging as an important component of emissions reduction policies, there is a need to demonstrate whether data collected by these programmes are robust enough to comply with international monitoring requirements and to improve these programmes where possible [12]. Historically, most natural resource monitoring has been conduct by experts; though some studies in the field of conservation biology have shown that non-experts can monitor data as reliably as experts [13, 14]. However, few studies have looked specifically at the quality of community forest monitoring programmes.

Recently, three studies have quantitatively assessed the differences in experts’ and non-experts’ field measurements of forest biomass stocks. The first study by Danielsen et al. [6] involved a post*-hoc* comparison of existing community-based forest biomass inventory to that of experts in India and Tanzania. They found no significant differences in the forest biomass estimates, an important preliminary demonstration of the quality of non-experts’ biomass surveys. The second study from Butt et al. [15] involved a more detailed analysis and an expert data check from a subsample of non-experts’ (volunteers) field measurements in the Oxford Forest, United Kingdom. Measurements were taken by both experts and non-experts of tree diameters and height, and from these results, a sampling error was produced. The study demonstrated that non-experts’ measurements had a greater sampling error than experts’ measurements. The third study by Danielsen et al. [16] was a resampling campaign of biomass inventories that compared tree diameter measurements and tree counts from experts and non-experts in Southeast Asia. The researchers found that though biomass estimates were generally similar, the estimates differed significantly in one-third of the sites. They also found that tree diameters were significantly different for half the measurements. These three studies demonstrated that non-experts’ biomass estimates and the field measurements required to calculate these estimates were generally of good quality. However, discrepancies did exist, and it remains unclear what types of error cause the largest discrepancies in forest biomass assessments.

Most of the countries participating in REDD+ activities are currently in the preparation phase, which involves the establishment of a national monitoring system [17]. Therefore, refining the accuracy of the data produced by community monitoring programmes is timely. Improving on the methodologies for data collection first requires the determination of the types of errors that are the most important, namely, which ones lead to the biggest discrepancies in forest biomass estimates. This study attempted to address this issue. We performed a full expert remeasurement campaign of community-led forest biomass inventories in the remote forests of Papua New Guinea. We compared 4,481 experts’ and non-experts’ measurements of tree diameter, height, numbers of trees and plot surface area. The study design allowed for the detection of errors in the field and how these contribute to discrepancies in forest biomass estimates. The study revealed unexpected results that could serve to improve forest biomass inventories and training protocols for experts and non-experts alike.

## Methods

### Ethics statement

The study was approved by the James Cook University Human Research Ethics Committee (HREC). Written consent was not sought nor required by the Ethics Committee because the study did not have the aim to assess the capacity of ‘individuals’ but instead of ‘programmes’ that employ local people. Prior to our undertaking the work, community meetings with local landholders were held. We, the scientists, relied on customary decision-making by the landholders for determining the duration and location of, and participants in the study. After a three-day training session with the participants, the participants were well informed and consented orally to the study. No minors participated in the study. The authority that issued the permit to work in the YUS (Yopno-Uruwa-Som) Conservation Area was the Tree Kangaroo Conservation Programm (TKCP).

### Study area

The study was conducted in the Saruwaged Range in Morobe Province, Papua New Guinea (PNG; 6°04’S, 146°48’E). This was the YUS Conservation Area, a region of 182,000ha covered by 70% primary forest ranging from 50masl to 3,100masl. The climate is perhumid, with a mean annual precipitation ranging between 2,600mm in the lowlands and 4,200mm in upper montane forests [18]. Mean annual temperature in the lowlands is 26°C, decreasing by about 5.4°C per 1,000m of elevation gain, reaching 10°C at 3,100masl. The assessment was carried out in three tropical forest types which fall in the high range for tropical forest biomass [19], ranging from c.a. 600Mg·ha^-1^ in the lowland coastal forest to 320Mg·ha^-1^ in upper-montane forest (unpublished data collected by authors).

### Participants

The local participants of this study were landholders who pledged land to the YUS Conservation Area. The pledged land formed the first and only protected area under PNG’s *Conservation Area Act 1978*, through the efforts of TKCP. About 30 villages with a population of ~12,000 are associated with the conservation area. The area features rugged topography and no road access, and the predominant livelihood is subsistence farming with a high dependence on forest resources. Only 1% of households in the area earned a monetary wage. In 2011, 40% of adults had never attended an educational institution, and those who did had attended for an average of six years [20]. Local participants for this study were chosen by committees of local landholders and were paid a standard wage set by the TKCP. To promote the exchange of knowledge, we requested that the teams consist of at least one person with traditional knowledge of the forest and at least one young person (< 25 years old). We formed three teams from three different communities, each consisting of six people from different language groups.

### Study design

Each team individually took part in a three-day training session aimed at teaching self-directed forest biomass inventory techniques. The training consisted of knowledge exchange on the role of forests in climate change, drill exercises and games with compasses, diameter tapes, clinometers, survey tapes and GPS, and random systematic site-selection techniques. The final day was dedicated to a full inventory ‘practice-site’ followed by a discussion on adaptive teaching and learning to address the most common mistakes and challenges. A fourth day of training involved only the team leader and aimed at standardising data collection.

Self-directed inventories by non-experts took place two weeks after the training. Forty-one plots were established, 14 plots from two of the teams and 13 from the third. In total, the study took 80 field days to complete between November 2010 and April 2012. Unassisted local teams conducted plot establishment and recorded the number of trees in the sample and measured their diameter and height. Local teams also collected the altitude and site coordinates with a GPS unit. The angle of the predominant slope, necessary for calculating the horizontal plot area, was recorded with a hypsometer [21]. These slope angle values were classified in one of the following categories: (≤ 11˚ = gentle), (12° to 25° = medium), (26° to 45° = steep) or (45° to 90° = very steep).

**Fig 1:**
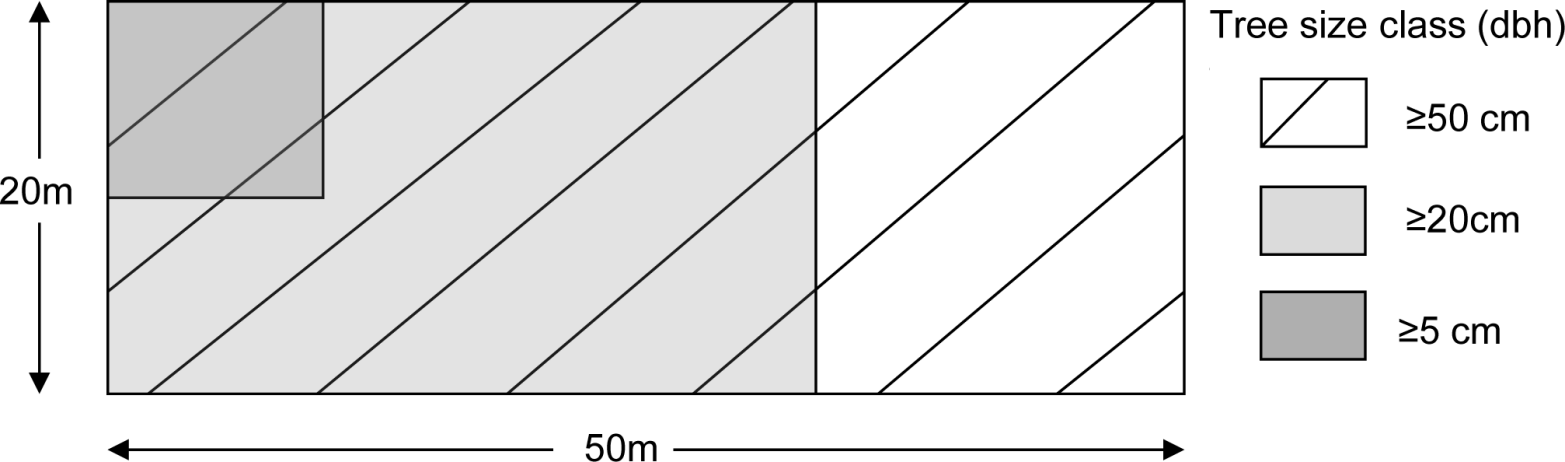
Diagram of a forest-biomass inventory plot (0.1ha) with subplots (0.06ha and 0.01ha).

Methods for plot establishment and estimating above-ground forest-biomass inventory were largely based on widely used LULUCF protocols [21] and tree height measurement protocols from Feldpausch et al. [22]. The plots (20m x 50m) were delineated using compasses and survey tapes (Fig 1). Trees were counted in appropriate subplots and tagged with a unique id. Diameters at breast height (DBH) were measured to the nearest millimetre with a diameter tape at 1.3m above the ground. Heights were measured by standing directly below the crown and measuring the highest point in the canopy with a rangefinder (LaserAce hypsometer) multiple times until the highest point was reliably identified. Wood Specific Gravity (WSG) was from standard values from the Asian rain forest dataset [23, 24, 25]. Tree species identification to determine WSG was done on botanical specimens by experts and by DNA-barcoding analysis of leaf samples collected in the field. Where possible, non-experts also recorded tree species’ names in the experts’ languages. For the site to be located for remeasurement by experts, the non-expert teams pegged the four corners of the plot markers; a GPS coordinate and the location description were also recorded. The southwest corner plot was selected using a random systematic technique, and sites were expected to have a minimum 120m distance from adjacent sites.

### Validation process

The 41 sites established and measured by communities (non-experts) were entirely remeasured by scientists (experts) using the same methodologies. Validation was performed in the same field-season after the community teams had finished their inventories, for a total of three field seasons. Experts worked in pairs, and a total of six experts participated in this study, and everyone had completed a degree in the natural sciences.

Both experts and non-experts delineated their own plot from the same starting point, however expert also remeasured the plot demarcation of the non-expert. The expert team verified the plot surface area set by the non-expert team by re-measuring the distance and angles of the four pegs demarcating the outer perimeters. Subplot areas were not verified. The plot surface area was calculated using a trapezoid formula with the four distances and four angles measured from the non-expert plots. The biomass estimates from all trees sampled within the boundaries of expert was compared to the biomass estimate from all trees sampled within the non-expert plot. To compare the tree counts by experts and non-experts, we only included trees the overlapping area of expert and non-expert plots. We acknowledge that by restricting the comparison to trees that were presumably accounted for by both observers, we are biasing the discrepancy of tree counts to those restricted to the overlapping area, however, because plot size varied and they may not entirely overlap, it was impossible to account for the effect of missing or extra trees outside the overlapping areas.

Our sample area had a total of 1,433 trees; 1,364 of these were in both datasets (expert and non-expert) and of these, 1,281 tree height measurements were recorded. Some trees could not be assessed for height because of visual obstructions. DBH were validated by experts using the same measurement point marked by the non-experts. Note that we did not validate WSG, the third parameter in the allometric model, as these values were obtained only by the expert team. For tree counts, trees were deemed to have been ‘missed’ by non-experts only when a tree within the designated plot had not been tagged and recorded by non-experts. However, trees were deemed ‘extra’ only if they had been tagged and recorded by non-experts but were deemed too small or too large for the subplot or if trees were recorded by non-experts but could not be located by the experts.

To estimate the dry biomass of trees, we used allometric equations for wet tropical forests formulated by Chave et al. [26]. We chose this equation because it performs well across a broad range of wet tropical forests using DBH, height and WSG parameters. Carbon values from biomass estimate could be derived using a factor of 0.5, but we reported only values in biomass (in kg or tonnes) (IPCC Guidelines).

### Statistical analysis

**Fig 2:**
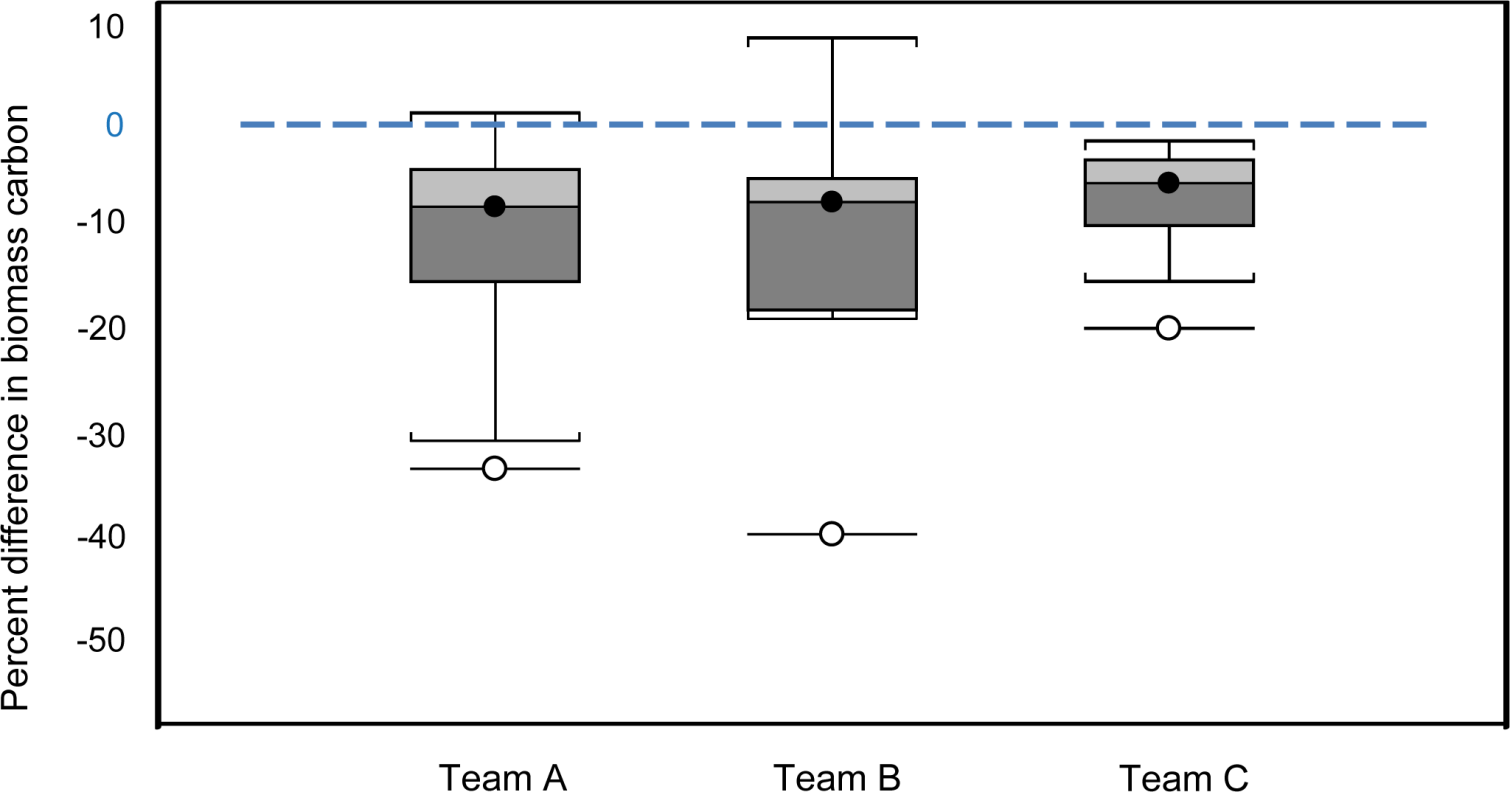
Percentage difference of biomass estimates of those produced by three non-expert teams (N = 41) and those produced by experts (N = 41). Team A and Team B had 14 participants, and Team C had 13 participants. The line across the middle of each box represents the median; the boxes show the interquartile range for around the median for half the data at the top and the other half at the bottom; the whiskers represent the 10^th^ to the 90^th^ percentile, and the outliers are demonstrated by the empty circles.

We tested for deviation from normality using Shapiro Wilk before testing pairs of measurements with parametric (t-test); DBH measurements (number of pairs = 1,364), height measurements (number of pairs = 1281) and plot surface area (number of pairs = 41) or non-parametric tests (Wilcoxon) for numbers of trees sampled at the site level (number of pairs = 41) and used Kruskal-Wallis to compare the final mean biomass estimated by the two teams at the landscape scale. We considered the three non-expert teams as one group because our aim was to compare non-experts with experts, and not to compare non-experts with themselves (Fig 2). However we did compare the effect of different teams on measurements with a Kruskal-Wallis and found no effect for DBH (χ^2^ = 0.98, df = 2, *P =* 0.61), plots surface area (χ^2^ = 1.4, df = 2, *P =* 0.50) and biomass (Fig 2, χ^2^ = 0.98, df = 2, *P =* 0.61) but did detect a significant effect on height (χ^2^ = 11.73, df = 2, *P =* 0.003).

To compare the expert and non-expert datasets, we used the Standard Major Axis (SMA) regression model because, as opposed to the Generalized Least Squares (GLS) regression, it does not assume that X is dependent on Y and that X is without error. However, we also report the analysis from the GLS regression as it is most commonly used in similar analyses [27, 28].

**Fig 3:**
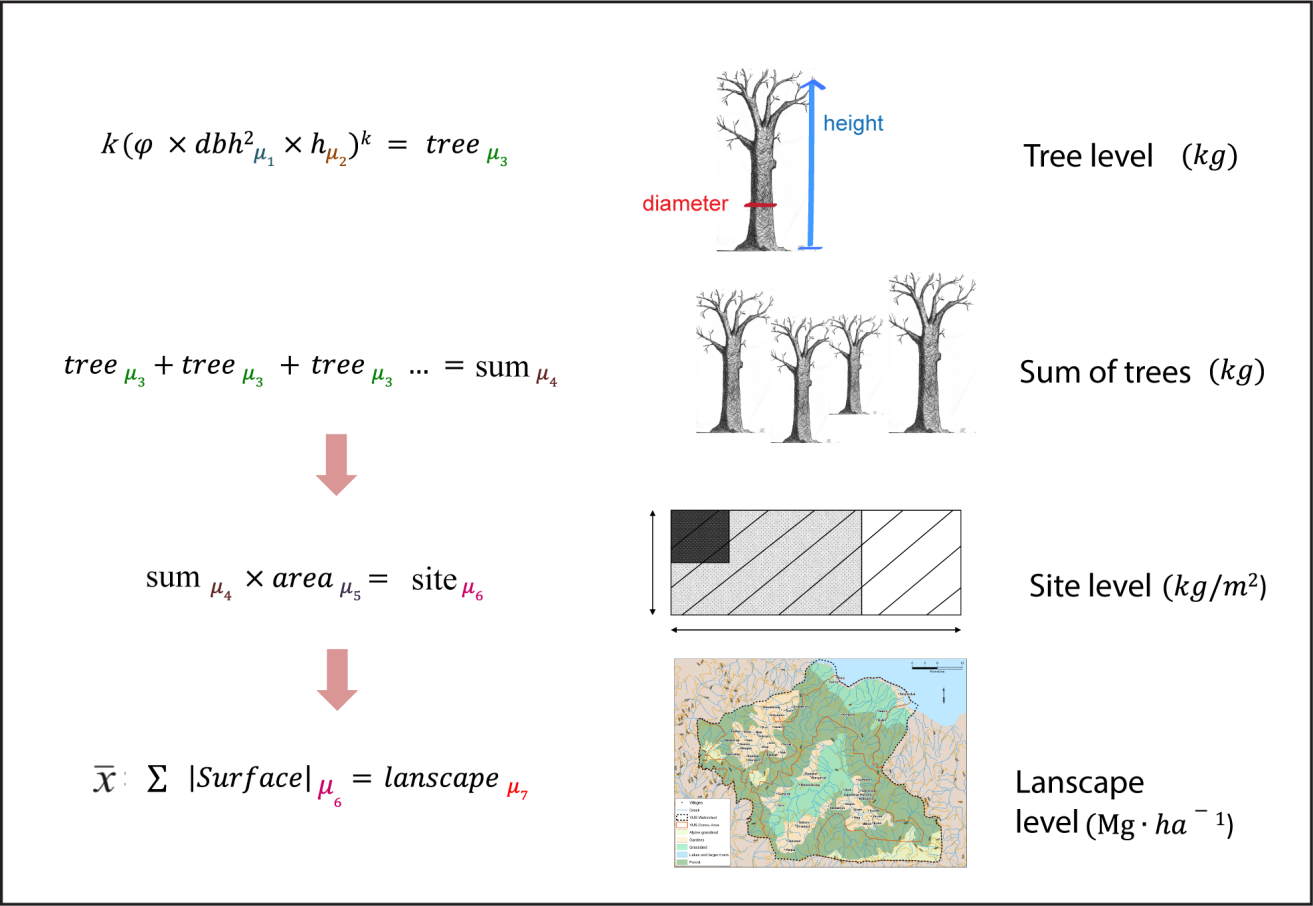
Schematic description of how errors (μ) propagate from the tree level to the landscape level in field biomass inventories.

We explored the influence of the discrepancy in DBH (% error) and height (% error) on discrepancy in tree biomass (% error) using a Generalised Linear Model (GLM) for trees that had both DBH and height measurements from expert and non-experts. We did not perform GLM at the landscape scale as assessing errors at this level required using plot averages for height and DBH which would have masked the variation from negative and positive values measured within the plot. Therefore, we took a hierarchical approach to determine what differences in measurement types from expert and non-expert measurements affected the overall differences observed in biomass (Fig 3). First we compared all pairs of DBH and height. Second, we calculated tree biomass (in kilogram) using expert derived measurement (DBH and H) and replaced only DBH with the value obtained by the non-expert and then calculated once more by replacing height only with the non-expert derived measurement, while always keeping WSG constant. In total, four biomass values were produced for each tree using Chave et al [26] allometric equation, with the combination of the following sets: 1) DBH_(expert)_ and H_(expert)_; 2) DBH_(non-expert)_ and H_(expert)_; 3) DBH_(expert)_ and H_(non-expert)_; 4) DBH_(non-expert)_ and H_(non-expert)._ Third, the differences in biomass of the sum of each set (1–4) were calculated for the 41 sites to determind the difference in kilograms associated with DBH and height; we only included trees that had expert and non-expert measurements for height. Forth, we quantified errors introduced from missing/extra trees. Fifth, we quantified errors introduced by plot surface area (1000m^2^). Finally we extrapolated biomass errors from each measurement type at the landscape level and compared the final estimates.

All statistical analyses were conducted in the S-PLUS Enterprise Developer Version 8.0.4 for Microsoft Windows: 2007 1988, 2007 Insightful Corp and R version 2.15.1 (2012-06-22) ‘Roasted Marshmallows’ 2012 the R Foundation for Statistical Computing.

## Results

### Biomass discrepancies

First we present the final summary of the errors identified through our hierarchal approach; then, in the following section, we explore in details the different error types for each field measurements to further understand why non-experts produced generally lower biomass values.

For the 41 site pairs, experts’ and non-experts’ biomass estimates were not significantly different (Wilcoxon signed-rank: n = 41, W = 1.21, *P* = 0.23) and were strongly correlated (GLS: F_1,39_ = 64.0, *P* < 0.001 R^2^ = 0.62). However, 78% of the non-experts’ biomass estimates were lower than those of the experts (Fig 2), resulting in a 9.1% difference between experts’ and non-experts’ biomass estimates at the landscape level.

**Fig 4:**
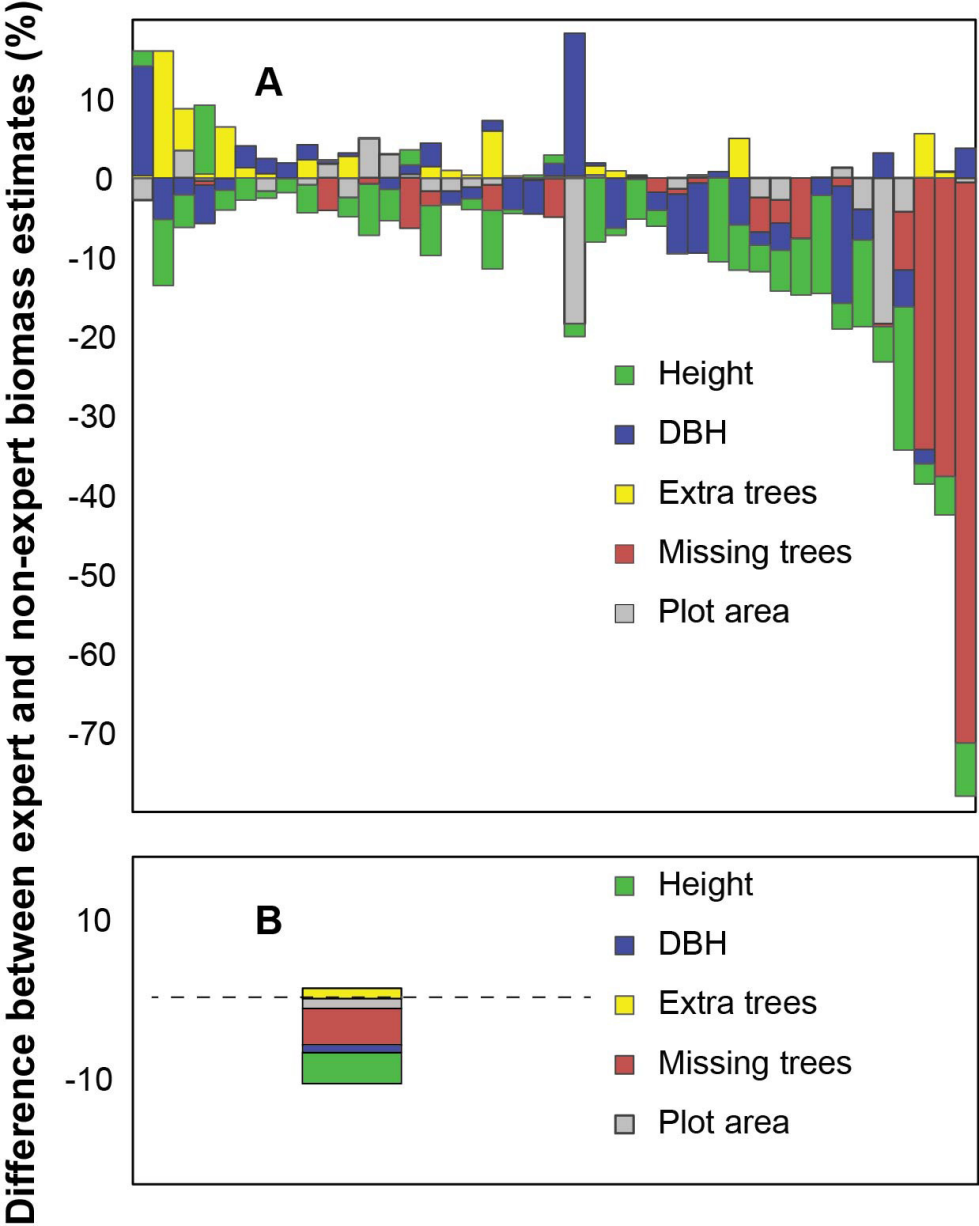
Difference in experts’ and non-experts’ biomass estimates due to measurement discrepancies at the A) site level and B) landscape level. Negative values represent smaller values of non-experts compared to experts.

Overall, we found that height measurements introduced the majority (71%) of site level biomass discrepancies (GLM, F = 2691, df = 1, 1057, R^2^ = 0.714, P < 0.0001), while the combined DBH and H biomass error ranged from +15% to –25% (Fig 4A). At the site level, discrepancies introduced from plot surface area and tree counts broaden the error range to +15% to –74% (Fig 4A). In order of importance, total discrepancies in biomass at the landscape level were caused from height (41.7%), missing trees (37.4%), plot surface-area (12.1%) and DBH (8.8%) (Fig 4B).

### Tree-level measurement

**Fig 5:**
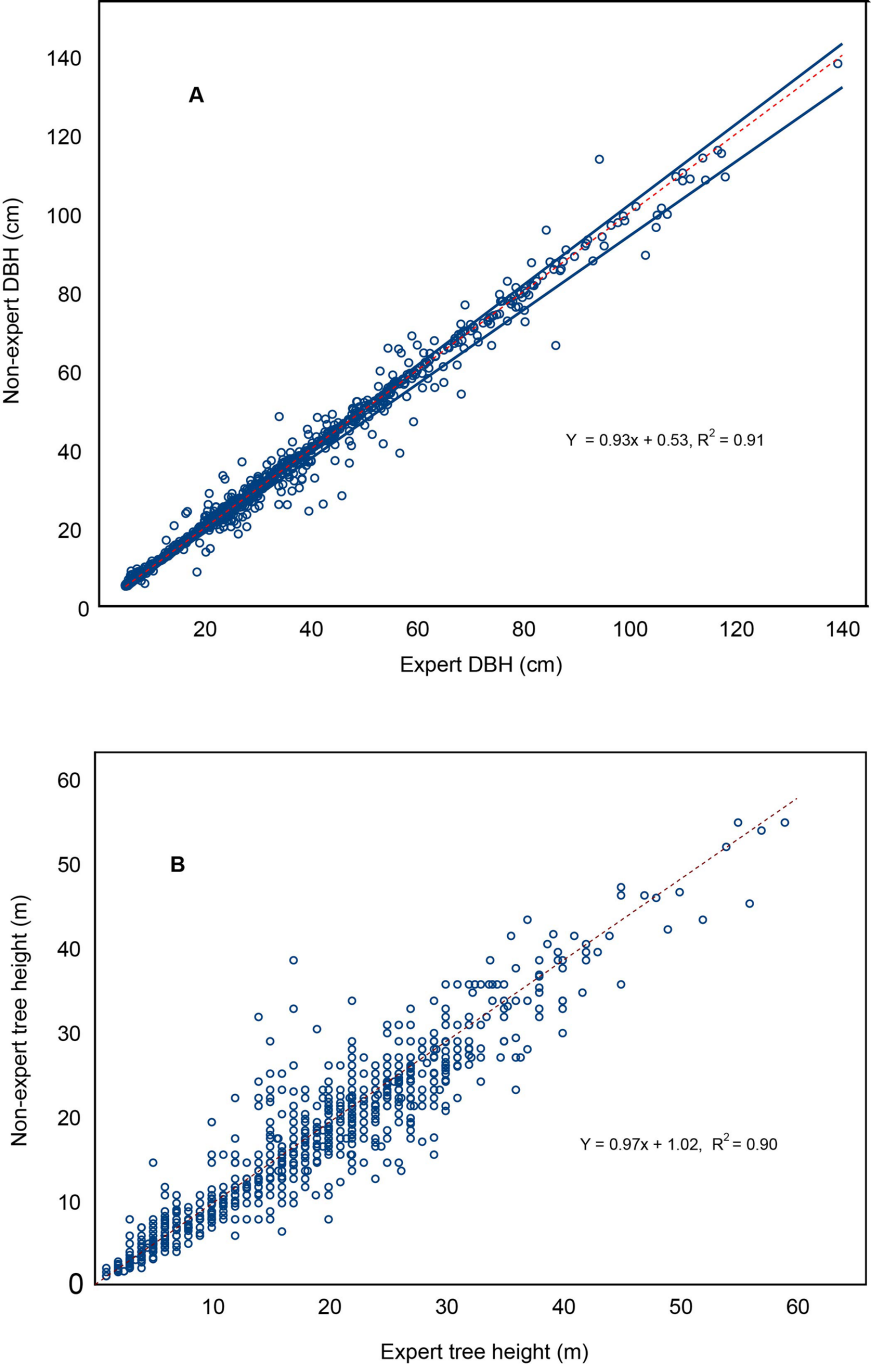
A) The relationship between expert and non-experts pairs of DBH (N = 1364) and B) the relationship between pairs of height measurements by expert and non-experts (N = 1281). A) The dotted red line represents a 1:1 relationship, the area between blue lines represents the 95% confidence interval, and the regression equation is from a GLM. B) The dotted red line represents a 1:1 relationship, and the regression equation is from a GLM.

There was no significant differences in experts’ and non-experts’ DBH measurements. More than a third of DBHs were exactly the same (Wilcoxon signed-rank: number of pairs = 1,364, W = 2.1, *P* = 0.07). Experts’ and non-experts’ DBH values were strongly correlated (S1 Table, Fig 5A, SMA R^2^ = 0.99, GLS R^2^ = 0.91) with a bias toward lower DBH in non-experts’ surveys indicated by slopes of regressions having values significantly lower than one (S1 Table, Fig 5A, SMA and GLS, *P* < 0.0001).

Height measurements were significantly different from experts’ and non-experts’ surveys (Wilcoxon signed-rank: number of pairs = 1,281, W = –10.2, *P* < 0.001). In particular, the proportional error in height was significantly larger for big trees, with 1.1% difference in measurement, which was about double the difference for smaller trees (Kruskal-Wallis chi-square = 64.9, df = 2, *P* < 0.001). However, the relationships between height pairs were strong (Fig 5B, SMA model: R^2^ = 0.92 and GLS model R^2^ = 0.90, S1 Table), with most pairs (98.4%) varying by less than ± 10% (Fig 5B).

Overall, most of tree level biomass discrepancies resulted from combined height and DBH measurement errors on large trees (DBH ≥ 50cm). Measurement errors on large trees caused 85% of the discrepancy; though these large trees consisted of only 14% of the stems. Whereas, combined DBH and height measurement error on small trees (DBH <20cm) had a negligible effect on tree biomass estimates, causing less than 1% discrepancy, outlining the importance of properly measuring large trees.

### Site-level measurement

**Fig 6:**
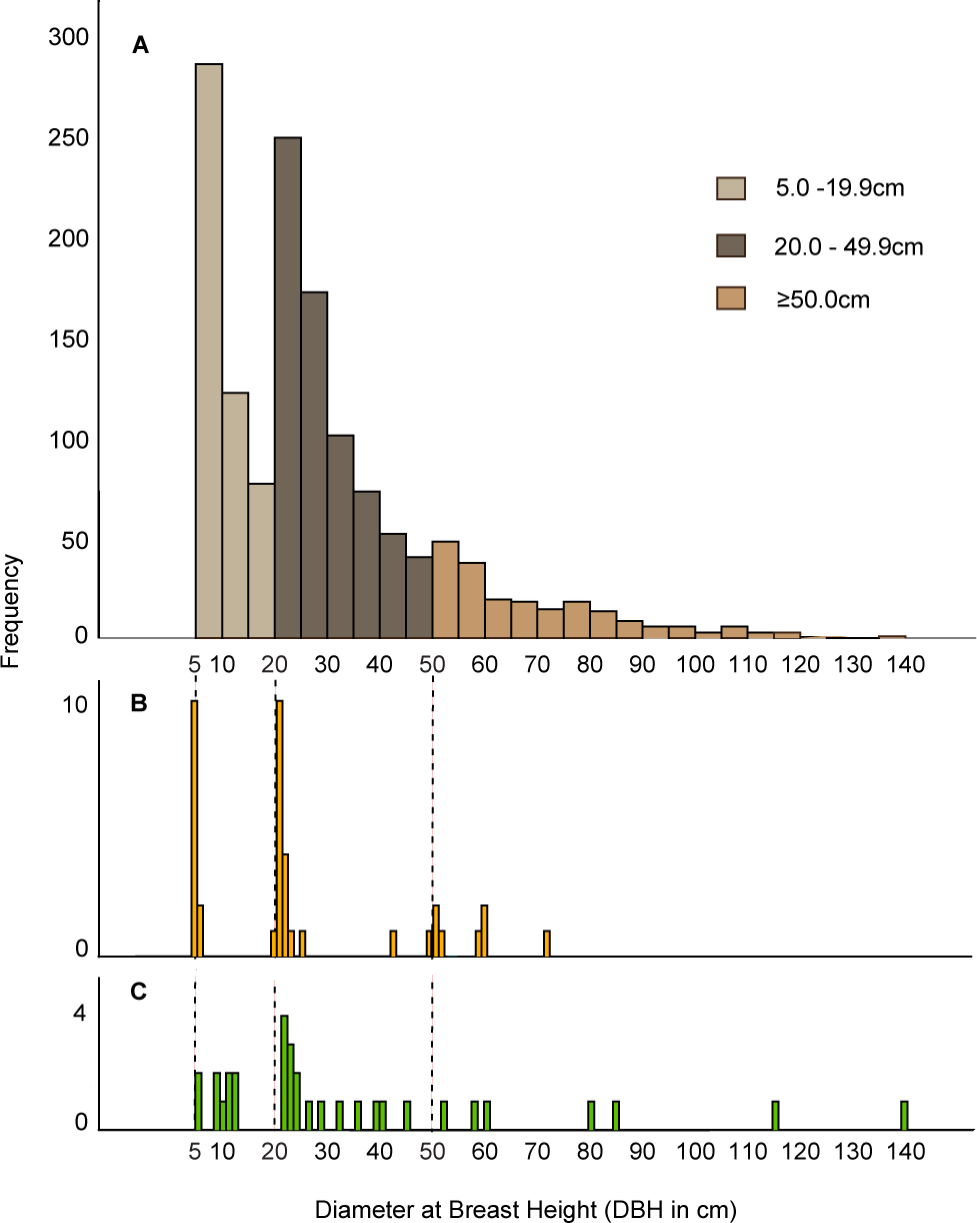
The size distribution of A) all trees, B) trees missed and C) extra trees in non-experts’ surveys. The dotted lines represent cutoff DBH sizes for subplots (5.0cm, 20.0cm, and 50.0cm) for the three subplots (Fig 2).

When comparing the experts’ and non-experts’ datasets, there were 32 extra trees recorded and 37 trees missing in the non-experts’ dataset. However, there was no statistical difference in the number of trees recoded in the experts’ and non-experts’ sample (t = 0.54, df = 40, *P* = 0.59). Because the missed trees were larger on average than the extra trees, DBH of 31.5cm ± SD 26.7 from the missing trees vs. 23.0cm ± SD 17.2 for the trees recorded in extra, this resulted in biomass underestimates from non-expert surveys. We found that the size distribution of trees recorded in extra in the non-expert datasets were clumped and mostly within a few centimetres of the cut-off tree sizes for subplots (e.g. 5cm, 20cm and 50cm, Fig 6A and 6B), indicating a systematic bias toward recording trees in the subplot that were beyond the size-cut-off limit. Whereas, the size distribution of the missing trees was similar to that of the whole sample, indicating that missing trees were more likely a random error (Fig 6A–6C).

The surface area of the plots established by experts and non-experts was not significantly different (Z = 5.6, df = 40, *P* < 0.0001). Differences were small, with more than half the plots within ±1% of the standard area; well within the suggested accuracy for biomass inventories [29]. Furthermore, non-experts met all site selection criteria. They established sites with a minimum of 120m distance from the nearest neighbouring site and recorded the GPS coordinates within the expected range of accuracy of the GPS instruments. The slope angle classification in experts and non-experts were the exact same, except for one site, where non-experts reported a ‘medium slope’ whereas experts reported a ‘steep slope’.

Finally, non-expert survey recorded local names for 91% of the trees, for a total of 552 different names; a clear illustration of the wealth of knowledge held by local landholders. Because the local classification system did not completely overlap with Linnaeus species identification (e.g. the same tree species may have had two local names based on the location where it grew), drawing conclusions about the reliability of the traditional plant taxa identification would have required further investigations which went beyond the scope of this study.

## Discussion

By fully remeasuring and isolating the effects of 4,481 field measurements, we demonstrate that programmes employing local people (non-experts) can produce forest monitoring data as reliable as those produced by scientists (experts). These findings corroborate those of Danielsen et al [7, 9], and then build on them by validating tree height measurement and plot surface-area measurements. Moreover, our study design enabled us to discover that missed trees in non-expert datasets are a large source of error in biomass estimates. Our results also demonstrated that the combination of errors in DBH and height measurements on large trees (DBH>50cm) formed the bulk of errors at the tree level. Though larger trees may require more effort to measure precisely, they generally store most of the carbon in tropical rainforest [30] and training should emphasize techniques to overcome these difficulties.

In general, the non-experts produced lower biomass estimates than non-experts’. Non-expert surveys had on average 9.1% lower biomass, equivalent to 55.2 (SE ± 24.0) fewer tonnes of biomass per hectare, for a total of 7.0 million tonnes fewer (SE ± 3.1) in the study area (unpublished data by the authors). These differences could have important financial repercussions for communities participating in carbon projects. Identifying what field parameters led to the discrepancies in biomass estimates was one of the aims of this study.

The lessons learn from our hierarchal approach to identifying sources of errors in biomass analysis were that the most frequent errors in the field may not necessarily led to the biggest discrepancy in forest biomass estimates [31]. Partly because DBH and height measurements can interact in the allometric model and errors from plot size and discordant tree counts can be introduced at landscape scale. The most unexpected result in this study came from discrepancies caused by discordant tree counts. The missing trees caused 37.4% of the differences between expert and non-expert biomass inventories. Because missing trees were on average smaller than extra trees, biomass values from “missing trees” were more than compensated by the number of “extra trees”.

At the tree level measurement in heights caused more discrepancies than DBH measurements. A large proportion of DBH (37.1%) matched exactly; this was similar to results reported by Danielsen et al [16]. These results are encouraging as DBH are almost always required for biomass inventories [32]. Also at the landscape scale, height measurements introduced the most discrepancy in biomass estimates (41.7% of the error). Heights are difficult to measure accurately, even for trained experts under the best conditions, [33]. Based on findings from other studies and considering the rugged conditions of this study area, we believe that the discrepancy between expert and non-expert height in this study where within acceptable range. The expected difference between the height measurements between two groups of experts is 10% [32] and our results showed that most of the pairs (98.4%) varied by less than this. Furthermore, form Butt et al [15] the average difference of two expert height measurements for the same tree was 2.8m for tree <35m tall, while the difference in our study was only 1.6m for trees <35m tall.

Because height introduces important error in biomass estimates and that height measurements are time consuming, monitoring programmes may opt to forgo taking height measurements [33, 34]. However, omitting height measurements from biomass inventories may cause a greater degree of inaccuracy. Studies have shown that omitting heights can cause discrepancies in biomass of 38% in lowland forests [35] and 50% at higher elevations [36]. Whereas, our results showed that height caused only 3.8% of the total errors in biomass estimates. Therefore, instead of omitting height altogether, alternative methods to estimate tree height should be considered. For example, height can be accurately estimated using deterministic models that predict height from the DBH or from LiDAR technologies [22, 37].

Our results showed that rectangular plots were established with a high level accuracy by non-experts, even in difficult terrain using previously unfamiliar equipment. Though many plot sizes and shapes exists for monitoring forest biomass surveys [21], monitoring programmes may consider more rapid ‘plotless’ surveys. Because trees near size class limit were included, even if they were too small, careful consideration should be taken before adopting plotless methods. Studies have shown that as the lack of a sample perimeter in a dense forest may intensify biases toward excluding or including trees close to the boundaries [38].

Our study had a number of limitations, notably our study would have benefitted from repeating the experts’ and non-experts’ measurements to create sampling error. This could have served as a reference to compare the difference we observed between the experts’ and non-experts’ surveys. However, logistic constraints associated with working in the remote forests of PNG for extended periods made this impossible. We partially addressed this by comparing the difference we observed between experts and non-experts to other studies that have quantified sampling error in expert DBH and height measurements [15, 32]. Furthermore, this study did not validate wood-specific gravity (WSG), the third parameter used to estimate tree biomass. Many studies have shown that wood-specific gravity can be effectively and accurately predicted with taxonomic information in such a way to minimize the introduction of errors into biomass estimates [31].

Effort to produce national forest biomass inventories for REDD+ programmes could represent unique opportunity to catalogue biodiversity and traditional knowledge. Although more than 500 local tree taxa were recorded in the study, assessing the reliability of these data went beyond the scope of this study. This study, however, introduces the potential for DNA-barcodes methodologies to be used with species identification in community-based forest monitoring as DNA-based approach requires only a small sample (1cm^2^) of leaf material preserved in silica which can be shipped via regular mail for analysis. Using DNA-barcodes for tree species identification in areas where taxonomy is poorly known has been shown to be effective and would be particularly useful in areas where time and capacity are limited [39].

Only 3% of forest-rich developing countries have the expert capacity to monitor changes in forest biomass stocks [5]. Our results, together with other studies [6, 15, 16, 40] help demonstrate that programmes that engage local people could address this capacity gap by providing quality data to support national monitoring programmes. Though decades of scientific research has advanced and refined expert-led forest biomass inventories [41, 42], the advent of community-based monitoring will most likely give rise to new challenges. Thus, developing reliable methodologies that remain flexible for local realities while meeting the needs of the participant will the key to the success of locally based monitoring programmes.

Finally, the contribution of knowledge from local communities could go beyond collecting data for biomass estimates toward deepening our understanding of the changes accruing in tropical forests [43]. Our finding that local communities were able to identify over 500 tree types is the tip of ecological information iceberg held by these communities. The scale and the consequences of changes in tropical forests can be understood only through long-term monitoring studies, to which local communities could be valuable contributors [44].

## Supporting Information

S1 Table Two models (GLS and SMA) that describe the relationship between experts’ and non-experts’ DBH and height measurements. Errors in height and DBH from each tree size category have markedly different contribution to discrepancies of forest biomass
(DOCX)
