# Supplementary material for: Validating Community-Led Forest Biomass Assessments
Source: PLoS One. 2015 Jun 30;10(6):e0130529. doi: 10.1371/journal.pone.0130529 (PMC4488351; doi:10.1371/journal.pone.0130529)
Supplement: S1 Table — Errors in height and DBH from each tree size category have markedly different contribution to discrepancies of forest biomass (DOCX) [file pone.0130529.s001.docx]

**Supporting Information**

**Table S1 Two models that describe the relationship between scientist and community height and DBH**. (Ð) X = expert values. Y = non expert values. GLS and SMA used the same data but estimated different slopes and slope intercepts because GLS slope is fit to minimise the vertical residuals (all error in the y axis) whereas in the SMS the slope is fit to minimise the perpendicular distances from each point to the line, accounting for error in both y and x dimensions.

| **Measurement** | **Regression model** | **Slope of best fit ^Ð^** | **Coefficient of correlation** | **S.E. for slope and intercept** | **Test against slope = 1** |
| --- | --- | --- | --- | --- | --- |
| DBH | GLS | (Eq. 3) Y = 0.93x + 0.53 | R^2^ = 0.91 | ± 0.008; ± 0.15 | p < 0.001 |
|  | SMA | (Eq. 4) Y= 0.99x + 0.01 | R^2^ = 0.99 | ± 0.003; ± 0.001 | p < 0.001 |
| Height | GLS | (Eq. 5) Y = 0.97x + 1.02 | R^2^ = 0.90 | ± 0.009; ± 0.17 | p < 0.001 |
|  | SMA | (Eq. 6) Y = 1.06x - 0.035 | R^2^ = 0.92 | ± 0.003; ± 0.008 | p < 0.001 |
